# A "Matron's Corner"

**Published:** 1887-09-10

**Authors:** 


### The Editor's Letter-Box.

[Our Correspondents are reminded that prolixity is a great bar to publication, and that brevity of style and conciseness of statement greatly facilitate early publication.]

### A "MATRON'S CORNER."

Sir,—In answer to matron's letter in The Hospital, September 3rd, I, for one, shall be not only pleased, but grateful to see a "Matron's Corner" started. Changes which often take place after leaving hospital, particularly if work takes you out of England, separate you from your hospital chums, and you try in vain to hear or find out those who so kindly and patiently pulled you up the hill of hospital work, and by kind words made you stick to the great and good work of nursing, when patience has been overtaxed and temper tried. I feel sure from experience that such a Corner will be most useful in more ways than one, as it will give us hints, and open friendly discussions with matrons of the present, past, and future.

Rugby. An Old Matron.

